# Toward Antibody Production in Genome-Minimized *Bacillus subtilis* Strains

**DOI:** 10.1021/acssynbio.4c00688

**Published:** 2025-02-27

**Authors:** Tobias Schilling, Rebekka Biedendieck, Rafael Moran-Torres, Mirva J. Saaranen, Lloyd W. Ruddock, Rolf Daniel, Jan Maarten van Dijl

**Affiliations:** † University Medical Center Groningen, Department of Medical Microbiology, University of Groningen, Hanzeplein 1, P.O. Box 30001, 9700RB Groningen, The Netherlands; ‡ Braunschweig Centre of Systems Biology (BRICS) and Institute of Microbiology, Technische Universität Braunschweig, Rebenring 56, 38106 Braunschweig, Germany; § Theoretical Biophysics, Humboldt-Universität zu Berlin, 10115 Berlin, Germany; ∥ Faculty of Biochemistry and Molecular Medicine, Protein and Structural Biology Research Unit, University of Oulu, Aapistie 7B, 90220 Oulu, Finland; ⊥ Institute of Microbiology and Genetics, Department of Genomic and Applied Microbiology, Georg-August-Universität Göttingen, Grisebachstr. 8, 37077 Göttingen, Germany

**Keywords:** Bacillus subtilis, antibody, scFv, scFab, genome reduction, secretion

## Abstract

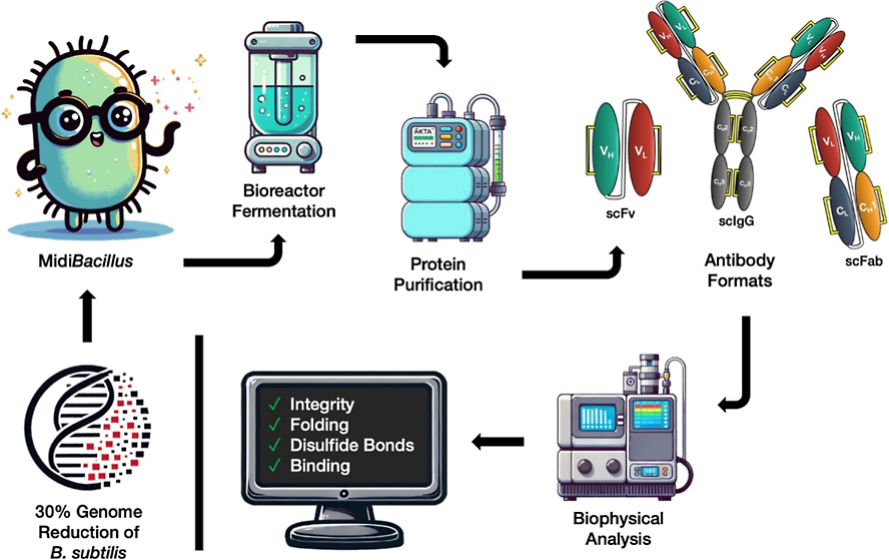

*Bacillus subtilis* is a bacterial cell factory with outstanding protein secretion capabilities that has been deployed as a workhorse for the production of industrial enzymes for more than a century. Nevertheless, the production of other proteins with *B. subtilis*, such as antibody formats, has thus far been challenging due to specific requirements that relate to correct protein folding and disulfide bond formation upon export from the cytoplasm. In the present study, we explored the possibility of producing functional antibody formats, such as scFvs and scFabs, using the genome-reduced *Midi*- and *MiniBacillus* strain lineage. The applied workflow included selection of optimal chassis strains, appropriate expression vectors, signal peptides, growth media, and analytical methods to verify the functionality of the secreted antibody fragments. The production of scFv fragments was upscaled to the 1 L bioreactor level. As demonstrated for a human C-reactive protein-binding scFv antibody by mass spectrometry, biolayer interferometry, circular dichroism, free thiol cross-linking with *N*-ethylmaleimide, and nano-differential scanning fluorimetry, *MidiBacillus* strains can secrete fully functional, natively folded, disulfide-bonded, and thermostable antibody fragments. We therefore conclude that genome-reduced *B. subtilis* chassis strains are capable of secreting high-quality antibody fragments.

## Introduction

The Gram-positive bacterium *Bacillus subtilis* and its close relatives have served as effective workhorses for producing technical enzymes at the industrial scale for more than one century.^1^ In 1922, the α-amylase “Rapidase” was the first commercialized enzyme produced with Bacillus, which revolutionized the industrial conversion from starch into sugar.^2^ In 1959, the first commercial detergent powder containing a protease produced by *B. subtilis* was marketed.^2,3^ Still, Bacillus is the dominating bacterial expression platform for large-scale production of enzymes, such as amylases, proteases, cellulases, esterases, xylanases, and phytases, which have become indispensable for food, feed, detergent, or paper production workflows. Bacillus’ outstanding ability to hypersecrete such a diverse spectrum of degradative enzymes is the outcome of adaptive evolution in its natural habitats with continuously changing physicochemical parameters and nutrient availabilities: predominantly the soil, plant rhizosphere, and intestinal tracts of mammals.^4−7^ By exploiting these capabilities of Bacillus in a biotechnological context and under optimized conditions, yields of over 25 g of protein per liter culture can be achieved.^8^

**Figure 1:**
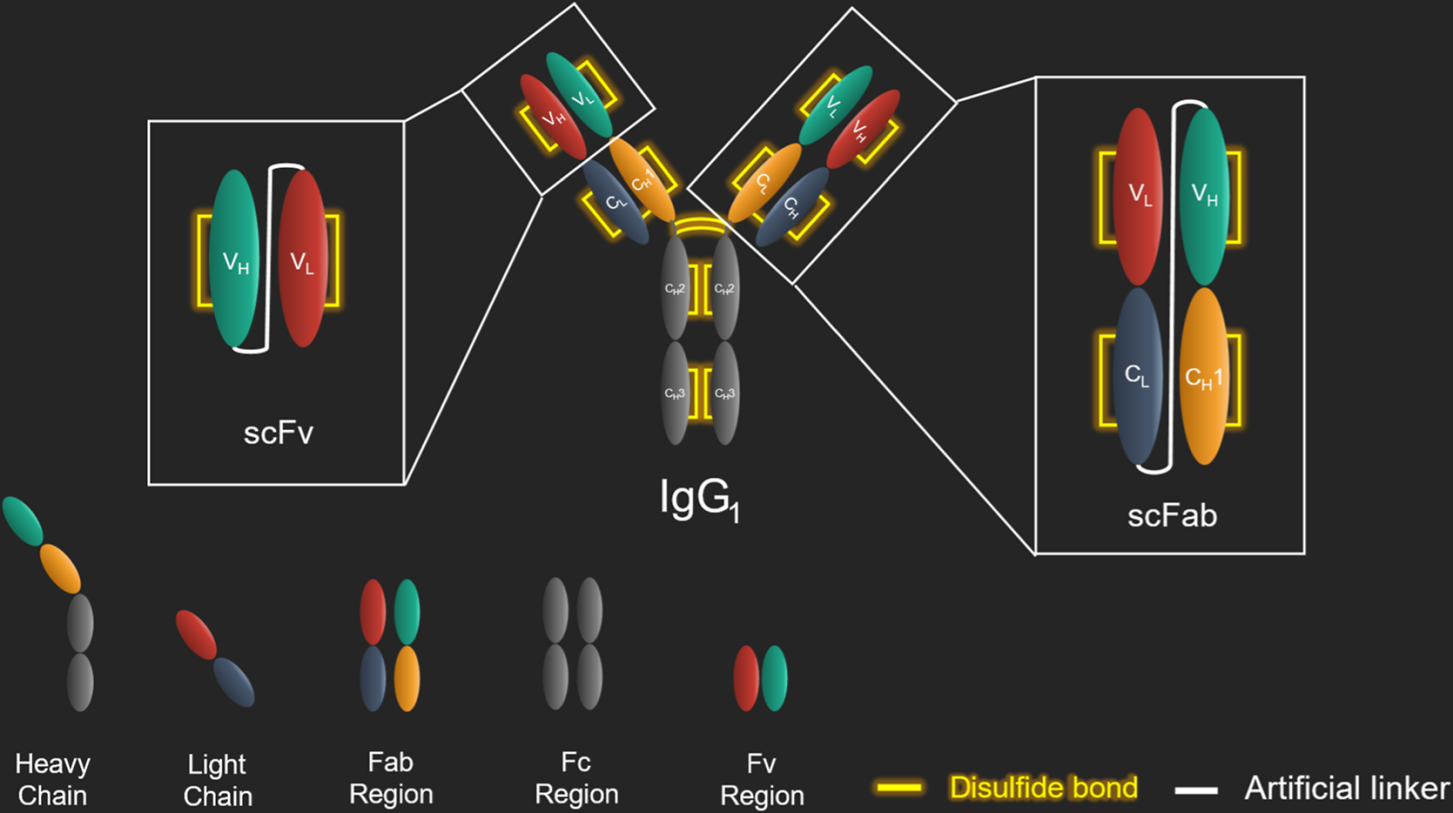
Different antibody formats derived from immunoglobulin G (IgG). Immunoglobulins, such IgG_1_, are composed of four polypeptide chains covalently linked via disulfide bonds,^19^ including two light chains (LC) and two heavy chains (HC). Each LC consists of one variable domain (V_L_) and one constant domain (C_L_). Each HC consists of one variable domain (V_H_) and three constant domains (C_H_1, C_H_2, and C_H_3). While the constant domains C_H_2 and C_H_3 of both HCs form the fragment crystallizable region (Fc region) that interacts with Fc receptors on the surface of immune cells, the V_H_, V_L_, C_L_, and C_H_1 form the fragment antigen-binding region (Fab region). Within the Fab domain, V_H_ and V_L_ form the Fv regions, which bind to the cognate epitope.^19^ Disulfide bonds preserve the structure and function of an antibody molecule: intradomain disulfide bonds stabilize the domains themselves,^20^ whereas interchain disulfide bonds connect both HCs, as well as each HC with its respective LC.^19^ The smallest functional IgG derivate is formed by a unimolecular fusion of V_H_ and V_L_ (V_H_–linker–V_L_) and generally referred to as single-chain Fv (scFv). scFvs cannot interact with Fc receptors but will bind target antigens.^21,22^ The next larger IgG format consisting of the Fab region domains is called a Fab antibody, which can exist as two molecules (Fab, V_L_–C_L_ and V_H_–C_H_1) or as one single-chain Fab (scFab, V_L_–C_L_–linker–V_H_–C_H_1).^21,22^ The linkers used to construct scFabs are rich in glycine and serine residues, granting structural flexibility to connect the different domains. In single-chain Fabs, the disulfide bond between the C_L_ and C_H_1 regions is dispensable.^21^ The smallest possible antibody format is a camelid heavy-chain variable domain fragment, also referred to as nanobody, but notably, a nanobody is not IgG-derived.^15,23,24^

In addition to industrial enzymes, another category of recombinant proteins with a massively higher retail price per unit has created a steadily growing market: biopharmaceuticals.^9^ Several pharmaceutically relevant proteins have so far been expressed in Bacillus in the context of academic studies but with noncompetitive product yields compared to other expression hosts.^10,11^ Antibodies (Figure 1) represent the currently most dominant category of biopharmaceutical proteins.^10^ However, the number of different antibodies expressed in Bacillus strains has remained low so far.^12−15^ The highest published antibody yield so far concerns the chicken-egg-lysozyme-binding D1.3 single-chain antibody (scFv), for which ∼120 mg of active protein per liter culture was obtained.^16^

One common feature of virtually all pharmaceutically relevant proteins, including antibodies, is the presence of disulfide bonds. These covalent bonds are in most cases important for the biological activity and structural stability of the respective proteins. Disulfide bonds are formed through oxidation of the thiol side groups of cysteine residues in an oxidative process that is catalyzed by thiol-disulfide oxidoreductases (TDORs) of the thioredoxin superfamily. TDORs that catalyze disulfide bond formation are encountered in the endoplasmic reticulum of eukaryotes and in extra-cytoplasmic compartments of bacteria. TDORs are also present in the cytoplasm of cells, but here, they catalyze disulfide bond breakage, as the cytoplasm is an overall reducing environment.^10,17^ Generally, disulfide bond formation represents a critical bottleneck for biopharmaceutical production in bacterial hosts, probably due to evolutionary constraints, since prokaryotes employ this post-translational modification less extensively than eukaryotes.^18^ Hence, Bacillus’ outstanding capabilities to produce industrial enzymes are usually not mirrored by an effective production of biopharmaceutical proteins of human origin.^10^

Generally, the yield of secreted proteins of interest (POIs) can be affected by several parameters. These include the number of gene copies per cell and the expression signals that drive transcription and translation of the POI,^25−28^ a proper match of the POI and the signal peptide (SP) that is used to direct the POI into the Sec pathway for secretion,^29,30^ the POI’s match with secretion pathway components and chaperones,^31−33^ the growth medium composition and culture conditions,^34^ and combinations of these parameters.^35,36^ While the massive secretion of exoproteases has originally been regarded as a positive trait of Bacillus, its downside is the potential degradation of POIs upon secretion. This is especially a problem in the production of those POIs that have been derived from distantly related microbial or eukaryotic species and that have not coevolved with the new Bacillus host to withstand its highly proteolytic extracellular environment. Since these exoproteases are dispensable under controlled growth conditions with optimal nutrient supply, the deletion of their genes has become a common practice in industrial strain optimization.^37,38^

**Figure 2:**
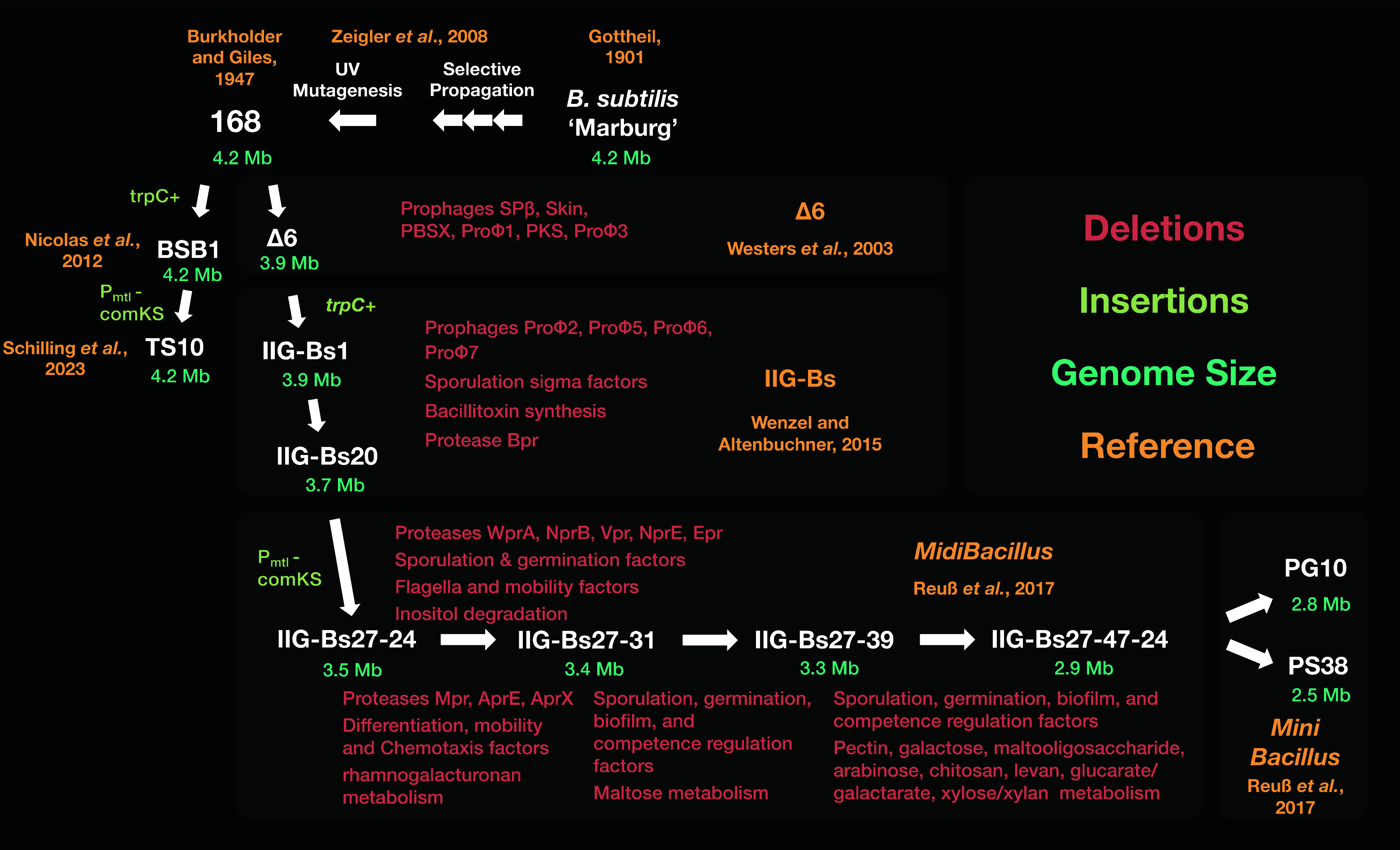
*MidiBacillus* and *MiniBacillus* strain phylogeny. The currently most genome-reduced *MiniBacillus* strains in the public domain, PG10 and PS38, were created in successive steps from the parental strain 168.^46,48,49^ The reference strain TS10 was constructed as a nongenome-reduced control strain, based on the strain BSB1.^50^ The original “undomesticated” ancestor of the strain line is the so-called Marburg strain.^51^ Several steps of “domestication” must have happened prior to the UV mutagenesis studies that led to the widely used 168 strain.^52,53^ The implemented genomic deletions in the currently available *MidiBacillus* and *MiniBacillus* strains are described in red, insertions in light green, corresponding genome sizes in cyan, and milestone descriptions in yellow.

Besides being an important cell factory, *B. subtilis* is one of today’s best-understood model organisms.^39^ Advances in omics analyses, genetic modification, and bioinformatics have allowed researchers to understand this bacterium as a molecular system but with many missing links still remaining.^40^ To simplify *B. subtilis* as a molecular system and to enhance protein production, large-scale genome reduction has been explored with positive outcomes.^41−45^ In particular, this has led to the *Midi*- and *MiniBacillus* strain line (Figure 2),^46−48^ of which some strains show significantly increased yields of secreted proteins that are considered as “notoriously difficult-to-produce”.^41,45^

In a recent study on the expression of disulfide-bonded proteins in *B. subtilis*, we discovered that, compared to the reference strain TS10 with a full-size genome, particularly the *MidiBacillus* strain IIG-Bs27-47-24 delivered a more than 3,000-fold increase in the secretion of active Gaussia Luciferase (GLuc), a protein containing five disulfide bonds that was derived from the bioluminescent copepod *Gaussia princeps*.^45^ On this basis, we hypothesized that genome-minimized *B. subtilis* strains might have a generally improved capacity for producing disulfide-bonded proteins. In the present study, we therefore explored the potential of the *MidiBacillus* strains for the expression of different antibody formats, including scFv, scFab, and scIgG, that target several different antigens.

## Results

### Screening Genome-Reduced *B. subtilis* Strains for scFv Production

**Figure 3:**
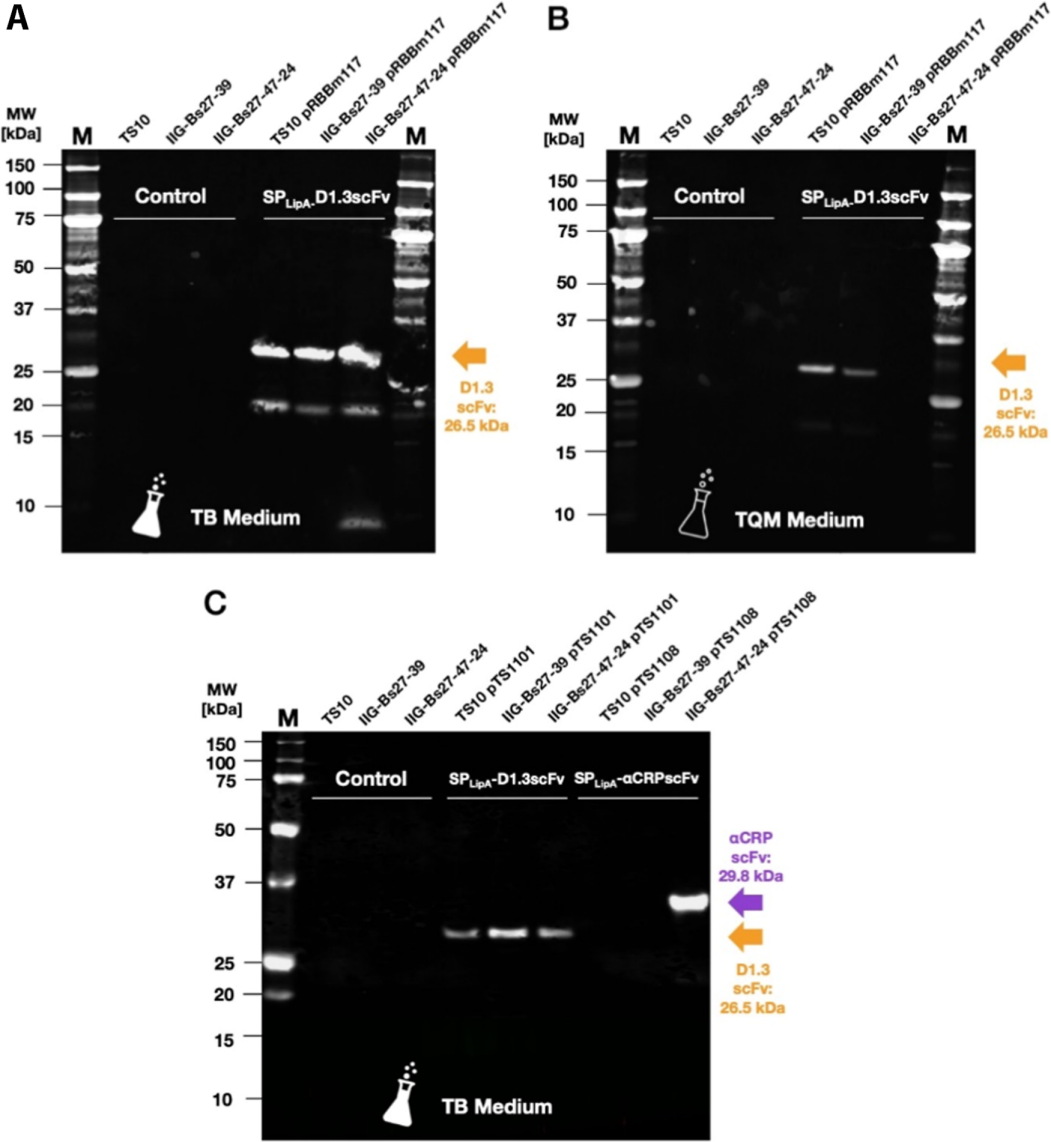
Secretion of D1.3 scFv and αCRP scFv by different *B. subtilis* strains. Representative Western blots of the extracellular protein fractions from different *B. subtilis* strains secreting D1.3 scFv or αCRP scFv. The presence of scFvs was visualized with a primary antibody against the C-terminally attached His_6_-tag. The colored arrows indicate the expected molecular size in kDa of the respective POIs. (A,B) Three different *B. subtilis* strains were benchmarked for secretion of the D1.3 scFv, using strains with or without plasmid pRBBm117. The bacteria were cultured either in the complex TB medium (A) or in the chemically defined TQM medium (B). Both Western blots (A,B) were processed and scanned simultaneously, to allow direct comparison of the D1.3 scFv levels. A higher exposed image of the blot in panel (B) is shown in Supplemental Figure S1. (C) Three different *B. subtilis* strains grown in TB medium were benchmarked for secretion of D1.3 scFv encoded by pTS1101 or αCRP scFv encoded by pTS1108. The respective plasmid-free strains were used for control.

To test which genome-reduced *B. subtilis* strain is most suited for the production of scFv fragments, genome-reduced strains IIG-Bs27-39 and IIG-Bs27-47-24, as well as reference strain TS10, were transformed with the plasmid pRBBm117. This plasmid encodes a fusion between the SP of a *B. subtilis* lipase (UniProt ID: Q79F14, codon-optimized; SPLipA) and the lysozyme-specific D1.3 scFv. Next, the strains were benchmarked for D1.3 scFv production upon growth in Terrific Broth (TB) or TQM medium. Both media were supplemented with xylose because expression of the SP_LipA_-D1.3 scFv construct encoded by pRBBm117 was driven by the xylose-inducible promoter P_*xylA*_.^54^ Furthermore, it should be mentioned here that the D1.3 scFv encoded by pRBBm117 and all other scFvs and scFab formats that were examined in this study harbored a C-terminal His_6_-tag to allow their detection with the aid of anti-His_6_ antibodies (Table 1). When cultured in the complex TB medium, all four strains expressed and secreted the D1.3 scFv in similar quantities, as was visualized by Western Blotting using an anti-His_6_ antibody (Figure 3A). Notably, two bands were detected, whereby the slower migrating band conformed to the expected D1.3 scFv size of 26.5 kDa, while the faster migrating band appeared at around 15–20 kDa. No protein bands were detectable with the anti-His antibody in the lanes with samples from plasmid-free control strains. Notably, the pRBBm117-carrying strains that were cultured in the defined TQM medium expressed significantly lower amounts of D1.3 scFv than did the same strains cultured in TB medium (Figure 3B). In fact, the IIG-Bs27-47-24 strain produced no D1.3 scFv at all when grown in TQM medium (Figures 3B and S1). The latter observation most likely relates to the fact that the IIG-Bs-27-47-24 strain lacks the β-xyloside permease gene *xynP*, which is required for uptake of xylose from the medium. Thus, while the P_*xylA*_ promoter is apparently active in complex TB medium, it presumably remained inactive in the chemically defined TQM medium, even when the inducer xylose was present.

**Table 1:** Strains, Plasmids, Recombinant Antibody Constructs, and Analytical Antibodies Used in This Study^a^

strain	genotype	phenotype	ref.
*B. subtilis* BSB1	*B. subtilis* 168 carrying the *trpC* gene from *B. subtilis* HVS495	tryptophan prototroph	(50)
*B. subtilis* TS10	168 *trpC* Δ*yvcA*::P_*mtl*_-*comKS*, 4.2 Mbp	prototroph, supercompetent	this study
*B. subtilis* IIG-Bs27-39	genome-reduced to 3.3 Mbp	relatively high biomass formation and growth rate	(47)
*B. subtilis* IIG-Bs27-47-24	genome-reduced to 2.9 Mbp	relatively low growth rate, unable to grow in most defined media, shows high secretion rates for some proteins	(47)

plasmid	relevant genotype and/or relevant characteristics	parental plasmid	ref.
pBSMul1_ SP_Epr+1__GLuc	SP_Epr+1_, Gaussia Luciferase (GLuc)	pBSMul1_GLuc	(45)
pPSPhoA5	SP-Pro_Lip_-PhoA (SP-Pro of *Staphylococcus hyicus* lipase, UniProt ID: P04635)	pPS2	(82)
pRBBm117	P_*xylA*_, SP_LipA_-D1.3 scFv-His_6_ (SP_LipA_ from *B. subtilis* lipase, UniProt ID: Q79F14, codon optimized)	p3STOP1623hp	(16)
pRBBm132	P_T7_, SP_LipA_-D1.3 scFabΔC-His_6_	pP_T7_	(83) and unpublished
pRBBm136	P_T7_, SP_LipA_-D1.3 scIgGΔC-His_6_	pP_T7_	unpublished
pRBBm230	P_*xylA*_, SP_LipA_-αCRP scFv-His_6_ (optimized gene)	pBC16	(84)
pTS1100	P_*Hpa*I*I*_, GLuc gene, Δ*cmR* Δ*bleO* (legacy resistance genes)	pBSMul1_SP_Epr+1__GLuc	this study
pTS1101	P_*Hpa*I*I*_, SP_LipA_-D1.3 scFv-His_6_	pTS1100	this study
pTS1102	P_*Hpa*I*I*_, SP-Pro_Lip_-PhoA	pTS1100	this study
pTS1103	P_*Hpa*I*I*_, SP-Pro_Lip_-D1.3 scFv-His_6_	pTS1101	this study
pTS1106	P_*Hpa*I*I*_, SP_LipA_-D1.3 scFabΔC-His_6_	pTS1101	this study
pTS1107	P_*Hpa*I*I*_, SP_LipA_-D1.3 scIgGΔC	pTS1101	this study
pTS1108	P_*Hpa*I*I*_, SP_LipA_-αCRP scFv-His_6_ (optimized gene)	pTS1101	this study
pTS1109	P_*Hpa*I*I*_, αCov1 scFv-His_6_	pTS1108	this study
pTS1110	P_*Hpa*I*I*_, αCov2 scFv-His_6_	pTS1108	this study
pTS1111	P_*Hpa*I*I*_, SP_WapA_-D1.3 scFab-His_6_	pTS1106	this study
pTS1112	P_*Hpa*I*I*_, SP_NprE_-D1.3 scFab-His_6_	pTS1106	this study
pTS1113	P_*Hpa*I*I*_, SP_Epr_-D1.3 scFab-His_6_	pTS1106	this study
pTS1114	P_*Hpa*I*I*_, SP_Bpr_-D1.3 scFab-His_6_	pTS1106	this study
pTS1115	P_*Hpa*I*I*_, SP_XynA_-D1.3 scFab-His_6_	pTS1106	this study
pTS1117	P_*Hpa*I*I*_, SP_LytF_-D1.3 scFab-His_6_	pTS1106	this study

expressed antibody fragment	antigen (UniProt ID)	antibody format	mol. weight [Da]	ref.
D1.3 scFv-His_6_	lysozyme (P00698)	scFv	26,474.24	(16)
D1.3 scFabΔC-His_6_	lysozyme (P00698)	scFab	50,372.45	(83)
D1.3 scIgGΔC	lysozyme (P00698)	scIgG	75,666.26	unpublished
D1.3 Pro_Lip_-scFv-His_6_	lysozyme (P00698)	Pro_Lip_-scFv fusion	49,333.99	this work
αCRP (LA13-IIE3)	human C-reactive protein (CRP, P02741)	scFv	29,801.97	(83)
αCov1 scFv-His_6_ (REGN10987)	SARS-CoV-2 spike protein (P0DTC2)	scFv	27,047.68	(85)
αCov2-His_6_ (REGN10933)	SARS-CoV-2 spike protein (P0DTC2)	scFv	27,064.94	(85)

Western blotting antibodies	specific binding target	type	source
Invitrogen PA1-181	*G. princeps* Luciferase	rabbit, polyclonal	Thermo Fisher Scientific
Invitrogen MA1-21315	His_6_ (HIS.H8)	mouse, monoclonal	Thermo Fisher Scientific
IRDye 800CW Goat anti-Rabbit IgG	rabbit IgG	goat, IRDye 800CW conjugated	LI-COR Biosciences, USA
IRDye 800CW Goat anti-Mouse IgG	mouse IgG	mouse, IRDye 800CW conjugated	LI-COR Biosciences

a (SP-Pro = signal peptide + propeptide).

To bypass the need for xylose induction, the sequences encoding the SP_LipA_-D1.3 scFv construct were cloned in plasmid pTS1100, a derivate of pUB110 with the constitutive P_*Hpa*I*I*_ promoter.^55^ This resulted in plasmid pTS1101. The same was done for the sequences encoding an scFv specific for the human CRP fused to the SP_LipA_ (SP_LipA_-αCRP), which resulted in plasmid pTS1108. The transformed strains were cultured in TB medium, either carrying no plasmid, pTS1101, or pTS1108, and secretion of the two scFv proteins was analyzed by Western blotting. All three tested strains carrying pTS1101 secreted the D1.3 scFv as observed for the pRBBm117-carrying strains, while the αCRP scFv was secreted exclusively by the IIG-Bs27-47-24 strain carrying pTS1108 (Figure 3C). Notably, the amount of αCRP scFv secreted by the IIG-Bs27-47-24 strain was substantially higher than the amount of D1.3 scFv secreted by this strain.

### Expression of scFvs, scFabs, scIgGs, and scFv Fusion Proteins

**Figure 4:**
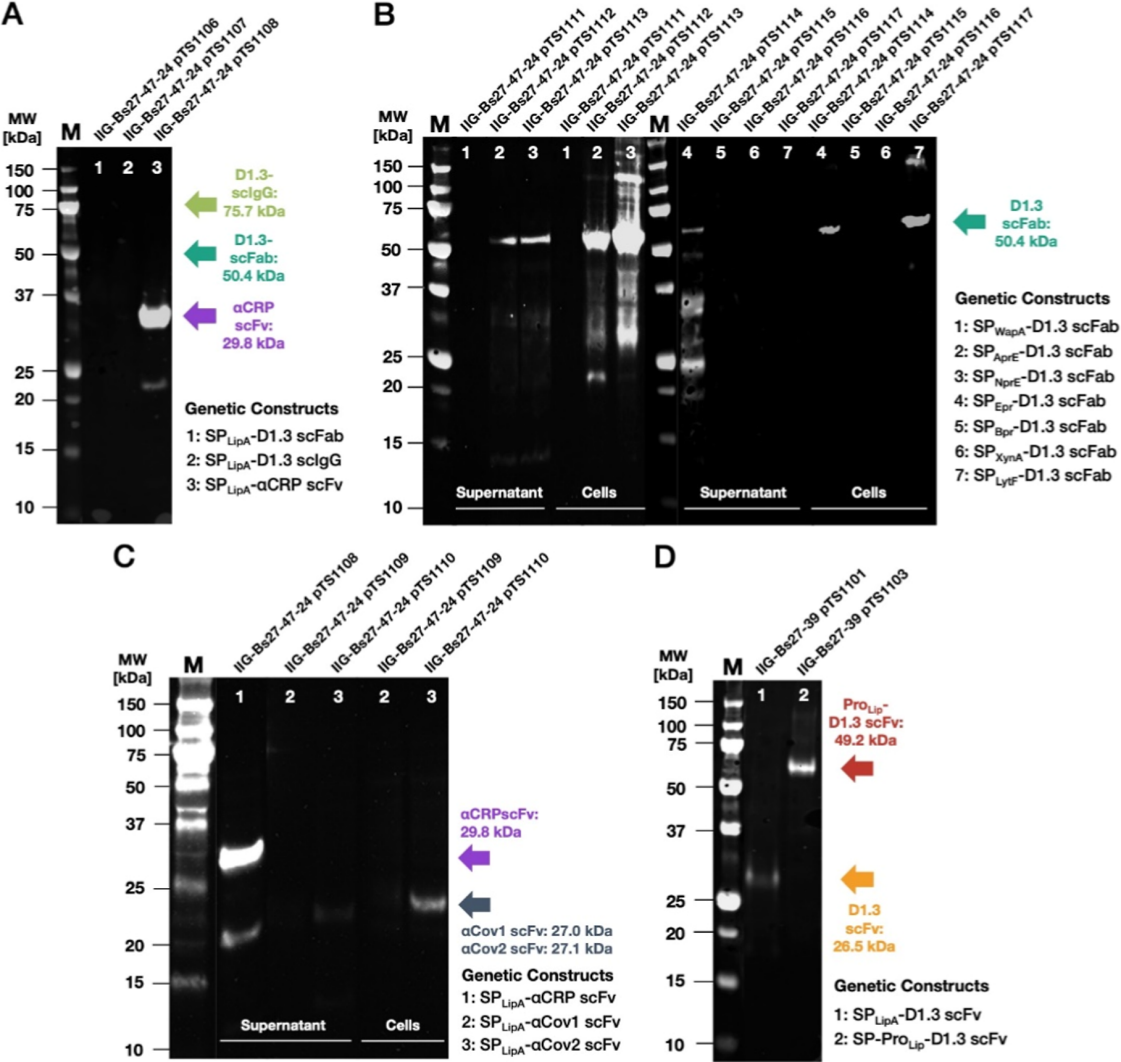
Secretion of D1.3 scFab, D1.3 scIgG, and a Pro_Lip_-D1.3 scFv fusion. Representative Western blots of different *B. subtilis* strains secreting scFv or scFab, immune-stained with a primary antibody against the His_6_-tag attached to the scFvs and the scFab, or with a secondary antibody against the D1.3 scIgG. The colored arrows indicate the expected molecular weights in kDa of the respective POIs. (A) D1.3 scFab, D1.3 scIgG, and αCRP scFv: the IIG-Bs27–47–24 strains expressing the respective constructs were grown in TB medium, and the proteins in their culture supernatants were analyzed. (B) D1.3 ScFab fused to different SPs from secreted *B. subtilis* proteins (i.e., SP_WapA_, SP_AprE_, SP_NprE_, SP_Epr_, SP_Bpr_, SP_XynA_, and SP_LytF_). The IIG-Bs27-47-24 strains expressing the respective constructs were grown in TB medium, and proteins in the culture supernatant and cellular fractions were analyzed. (C) αCov1 scFv and αCov2 scFv: the IIG-Bs27-47-24 strains expressing the respective constructs were grown in TB medium, and proteins in the culture supernatant and cellular fractions were analyzed. (D) Secretion of D1.3 scFv mediated by SP_LipA_ and SP-Pro_Lip_ both expressed in *B. subtilis* IIG-Bs27-39. Proteins in the culture supernatant fraction were analyzed.

Since *B. subtilis* IIG-Bs-27-47-24 was identified as the overall best-performing production strain for scFvs, and the plasmid pTS1100 enabled constitutive expression of target genes from the P_*Hpa*I*I*_ promoter, we decided to use this strain and plasmid for the expression of additional antibody formats (Table 1). However, while the D1.3 scFv (Figure 3A–C) and αCRP scFv constructs fused to SP_LipA_ were secreted well (Figures 3 and 4A), this was unfortunately not the case for the D1.3 scFab or the D1.3 scIgG fused to SP_LipA_ (Figure 4A).

To test if an exchange of the SP would lead to increased secretion of the D1.3 scFab, we constructed further SP fusion constructs using the SPs of the *B. subtilis* protein WapA (UniProt ID: Q07833), AprE (UniProt ID: Q03025), NprE (UniProt ID: P68736), Epr (UniProt ID: P16396), Bpr (UniProt ID: P16397), XynA (UniProt ID: P18429), and LytF (UniProt ID: O07532)^56^ (Figure 4B). While low amounts of secreted scFab were detected in the culture supernatant of the strain IIG-Bs27-47-24 expressing this antibody format fused to the SPs of AprE, NprE, or Epr, the scFab amounts detected in the corresponding cellular fractions were several folds higher. In the case of the SP_Epr_ fusion, the majority of the secreted scFab turned out to be degraded. This shows that the scFab was properly expressed but inefficiently folded into a protease-resistant conformation upon export from the cytoplasm.

While no expression of αCov1 scFv was detectable by Western blotting, αCov2 scFv was expressed in low amounts compared to αCRP scFv (Figure 4C). Thereby, a significantly more cell-associated unprocessed precursor of the αCov2 scFv fused to the SP_Lip_ was detected in the bacterial cells compared to the secreted form in the culture supernatant. This indicated that the SP_LipA_ mediated a basic but low level of αCov2 scFv secretion.

Lastly, we tested secretion of the D1.3 scFv construct fused to the SP-Pro_Lip_ peptide of *S. hyicus* (UniProt ID: P04635), which yielded higher levels of secreted D1.3 scFv-His_6_ compared to the expression of the SP_LipA_-fused D1.3 scFv construct (Figure 4D). Of note, a band with a size around 50 kDa was detected but no smaller band with the size of the mature D1.3 scFv, indicating the absence of Pro_Lip_ cleavage.

### Bioreactor Cultivation and scFv Purification

**Figure 5:**
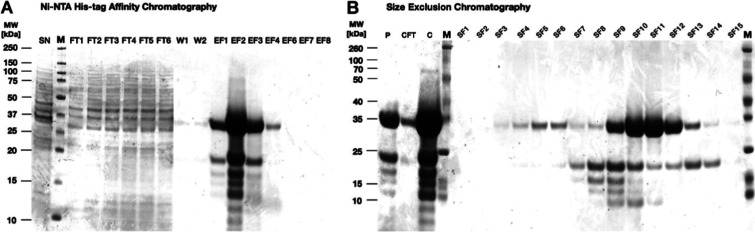
Bioreactor-scale production and purification of αCRP scFv. (A) Coomassie-stained LDS-PAGE of secreted proteins in the culture supernatant (SN) collected from the bioreactor at *t*_24h_, and 15 μL aliquots of all collected fractions from the subsequent Ni-NTA affinity chromatography purification of the (His_6_-tagged) αCRP scFv. (B) Coomassie-stained LDS-PAGE of the pooled (P) elution fractions EF1–4 obtained from the Ni-NTA affinity chromatography, the flow-through from the concentrator column (CFT) used for concentrating these pooled fractions, the concentrated pool of fractions (C), and all collected fractions from the size exclusion chromatography (SF; 15 μL each).

To upscale the scFv production from the 20 mL shake flask scale to the 1 L bioreactor scale, the strain IIG-Bs27-47-24 carrying plasmid pTS1108 (SP_LipA_-αCRP scFv-His_6_) was used. For this purpose, we explored the use of ABB^+^ medium because it mimics industry-scale fermentation conditions. Hourly samples were taken at the maximal cell density of the culture (OD_600_ ≈ 10), which was the case between 21 and 24 h of cultivation. The culture was harvested after 24 h of fermentation, and the (His_6_-tagged) αCRP scFv from 450 mL of freshly prepared cell-free culture supernatant was purified via Ni-NTA His-tag affinity chromatography. In addition to the main band with a molecular weight of 29 kDa, further smaller copurified fragments of around 15–20 kDa were detected upon LDS-PAGE and gel staining with Coomassie (Figure 5A). Subsequently, the elution fractions containing αCRP scFv were pooled, concentrated, and further purified via size exclusion chromatography (Figure 5B). Thereby, the fragments with lower molecular weight could largely be excluded. On this basis, fractions 9–12 were pooled and the other fractions were discarded. Per L of culture medium, approximately 6.6 mg of pure αCRP scFv was obtained.

### Biophysical Analysis of αCRP Secreted by *B. subtilis*

**Figure 6:**
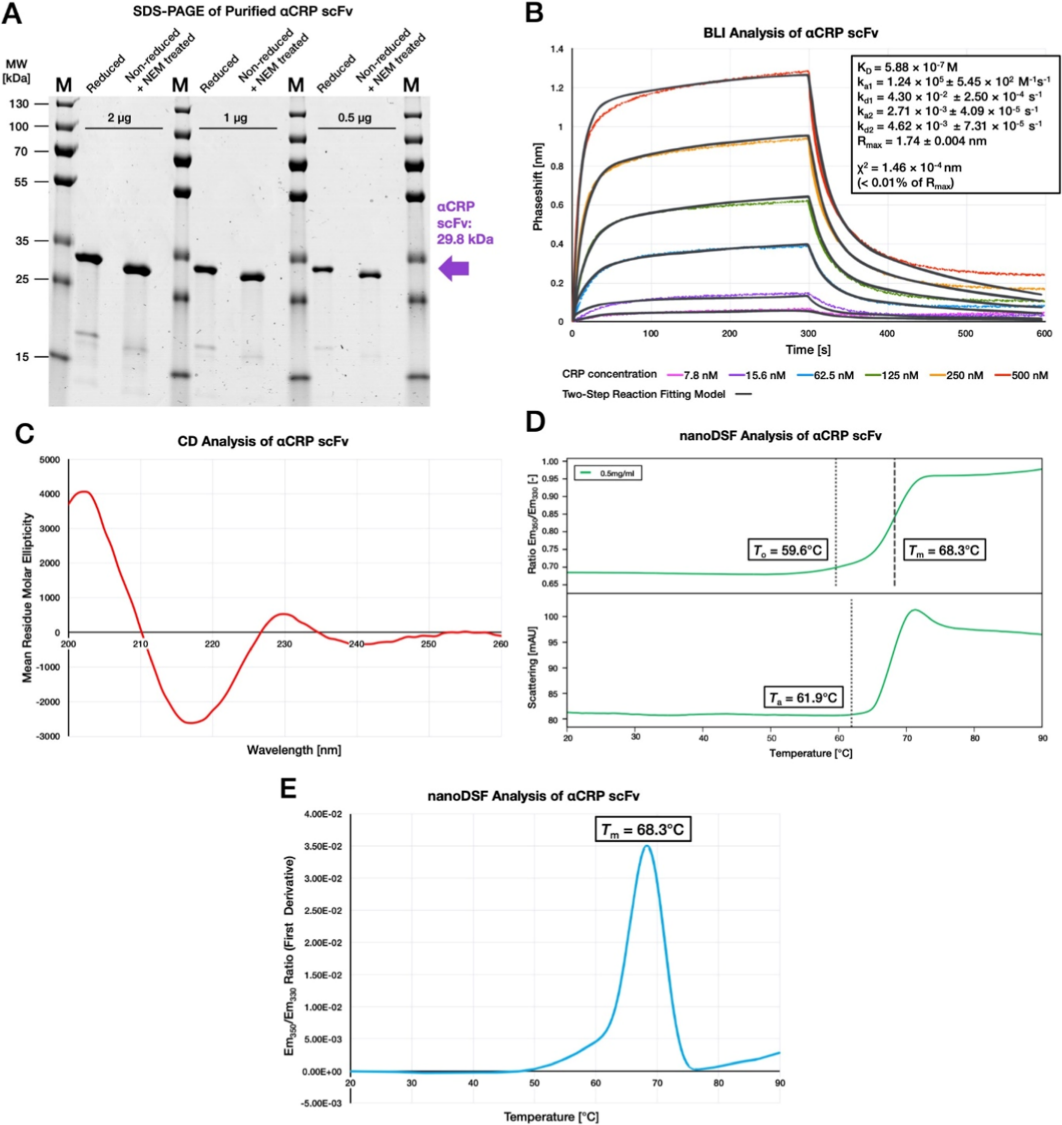
Biophysical analysis of αCRP scFv secreted by *B. subtilis*. (A) Samples of αCRP scFv were analyzed via SDS-PAGE, either in the reduced state or in the nonreduced state and treated with NEM. Main products of αCRP scFv appear homogeneously within the expected size ranges. In addition, a small amount of a putative C-terminal cleavage product of the scFv with a size of ∼15 to 17 kDa is detected. (B) Binding kinetics of the αCRP scFv and the respective CRP antigen was analyzed by biolayer interferometry (BLI) using different CRP concentrations as indicated. The phase shifts resulting from CRP binding to the immobilized αCRP scFv are plotted as a function of the exposure time. Using a two-state reaction fitting model, a dissociation constant *K*_D_ = 5.88 × 10^–7^ M was determined. (C) CD spectrum of the purified αCRP scFv. The main molar ellipticity as a function of the measured wavelengths indicates the proportional folding state of the protein. The spectrum shows that the purified αCRP scFv does not include a helical structure, as expected for antibody fragments. (D) nanoDSF thermogram. The upper panel shows the Em_350_/Em_330_ ratio, revealing the onset point for thermal degradation *T*_0_ = 59.6 °C. The light scattering thermogram in the lower panel shows the attenuation of the backscattered light intensity passing through the sample as a function of the temperature, indicating the onset point for thermal aggregation *T*_a_ = 61.9 °C. (E) First derivation of the Em_350_/Em_330_ ratio as a function of the time, with the inflection point indicating the *T*_m_ = 68.3 °C at which 50% of scFv is denatured.

The purified His_6_-tagged αCRP scFv obtained from bioreactor cultivation was analyzed for correct disulfide bond formation, native folding, antigen binding affinity, and thermostability. To check for disulfide bond heterogeneity, different amounts of the purified scFv were separated by SDS-PAGE gel in the reduced state and in the nonreduced state upon treatment with *N*-ethylmaleimide (NEM). Subsequently, the gel was stained with Coomassie brilliant blue (Figure 6A). Redox homogeneity and the absence of disulfide linked multimers were evident from the single αCRP scFv band detected for the NEM-treated, nonreduced sample. Next to the full-size αCRP scFv band, we observed faint additional bands with a size of around 15–20 kDa, which relate to degradation products of the scFv that were copurified with the full-size protein (Figures 4A and 5B).

To verify proper disulfide bonding of the purified αCRP scFv, electrospray ionization mass spectrometry combined with liquid chromatography LC-ESI-MS was performed. Here, a mass of 29,797.97 Da was expected for the disulfide-bonded αCRP scFv-His_6_, which is based on the theoretically calculated mass of 29,801.97 Da minus 4 Da due to the loss of 4 H^+^ by disulfide bond formation. In the case of one added NEM molecule per free thiol, an additional mass of 125 Da was expected. According to the detected masses, the fractional abundance of all molecules with the expected sizes (Table S2) summed up to around 90% (92.31% fractional abundance for the denatured sample and 89.15% fractional abundance for the denatured and NEM-treated sample). Thereby, masses of hydrated and dehydrated versions of the scFv were included, as hydration or dehydration is commonly observed during the electrospray ionization.^57^ Furthermore, low-abundant masses of a truncated C-terminal scFv fragment (*M*_theor_ = 16,331.89 Da) were detected (3.00% fractional abundance for the denatured sample and 1.91% fractional abundance for the denatured and NEM-treated sample). The molecular weight of these fragments observed by LC-ESI-MS, in combination with the observation of a smaller His_6_-tagged protein band on the SDS-PAGE (Figures 5 and 6A), verified the presence of a residual C-terminal product of the αCRP scFv that had resulted from cleavage between the amino acid residues Ser121 and Ser122 in the GlySer-rich linker sequence connecting the V_H_ and the V_L_ domains. In the NEM-treated sample, only one corresponding mass containing one additional thiol-linked NEM molecule was detected, with a fractional abundance of 0.94%. Accordingly, the combined LC-ESI-MS data imply that 99% of the detected αCRP scFv was correctly disulfide-bonded.

A BLI analysis was performed to measure the interaction of the αCRP scFv with purified CRP. This resulted in kinetic curves (Figure 6B) for which several different fitting models were tested. The best result delivered a two-step reaction model, resulting in a *K*_D_ of around 6 × 10^–7^ M and a χ^2^ ≈ 1.5 × 10^4^ nm, which is <0.01% of *R*_max_.

The secondary structure of purified αCRP scFv was analyzed by circular dichroism spectroscopy (CD). This showed that, as expected, the purified protein included no α-helical structures. Instead, this analysis revealed 47% beta-sheet, 13% turn, and 40% other structures (Figure 6C). From the acquired data, the van’t Hoff enthalpy (Δ_r_*H*^⊖^_1_ = 509.3 ± 10.4 kJ/mol) and a melting point 1 (*T*_m_ = 66.8 ± 0.1 °C) were calculated.

To verify the thermostability of the purified αCRP scFv, it was further analyzed via nano-differential scanning fluorimetry (nanoDSF), whereby the onset point for thermal degradation *T*_0_ was determined at 59.5 °C, and the onset point for protein aggregation at 61.9 °C (Figure 6D). The first derivation of the ratio between Em_330_ and Em_350_ was plotted as a function of the temperature, whereby its inflection point indicated that the *T*_m_ = 68.3 °C (Figure 6E).

## Discussion

In this study, different genome-reduced *B. subtilis* strains, expression systems, SPs to facilitate secretion, and growth media were tested for the secretion of various antibody formats, including scFv, scFab, and scIgG. The best-secreted antibody format, αCRP scFv, was expressed at the 1 L bioreactor scale, purified, and biophysically characterized.

A first round of strain screening identified the genome-reduced *B. subtilis* IIG-Bs27–47–24 strain as the best-performing strain in the secretion of the majority of scFvs tested. A direct comparison of the secretion levels of D1.3 scFv and αCRP scFv showed that different scFv molecules impose different demands on their production host. In this respect, it is noteworthy that the absence of exoproteases from genome-reduced *MidiBacillus* strains was not the only decisive criterion, since D1.3 scFv was also detected in the culture supernatant of the producing TS10 strain, which contains the full complement of *B. subtilis* protease genes. This implies that D1.3 scFv is secreted by TS10 and stable in the culture medium of this strain. In addition to the absence of eight extracellular proteases and one intracellular protease,^58^ other possible reasons for the superior performance of the IIG-Bs27-47-24 strain in the production of scFvs might be (i) this strain’s previously documented increased capacity for translation,^59,60^ (ii) elevated levels of secretion machinery components and chaperones for protein export via the Sec pathway, including the signal recognition particle components Ffh and FtsY, the protein translocation motor SecA, the signal peptidase SipS, and the extracytoplasmic peptidyl-prolyl cis–trans isomerase (PPI) and chaperone PrsA,^59,60^ (iii) an increased capacity for disulfide bond formation as exemplified with the five disulfide-bonds-containing GLuc protein,^45^ (iv) elevated levels of the quality control proteases HtrA and HtrB that also catalyze protein folding,^61^ and (v) redirected secretion stress responses that imply that the bacteria perceive less stress stimuli from heterologous protein production compared to the parental strain 168.^45,59,60^ However, the reason why the IIG-Bs27-47-24 strain shows an increased capacity for disulfide bond formation, as previously exemplified with GLuc,^45^ is presently not clear. We have previously shown that the secretion of active GLuc by genome-reduced bacteria was only partially dependent on the major thiol-oxidases BdbC and BdbD of *B. subtilis*.^45^ Yet, how substantial amounts of active fully disulfide-bonded GLuc can be secreted by a strain lacking both the *bdbC* and *bdbD* genes still needs to be elucidated. In any case, the latter observation suggests that so far unidentified thiol-oxidizing mechanisms, which function in parallel with the BdbC-BdbD pathway, may be active in the IIG-Bs27-47-24 strain.

Our results show that protein expression driven by the *xylA* promoter is not effective in the IIG-Bs27-47-24 strain, most likely because it lacks the main uptake system for xylose. This problem was bypassed by scFv expression from the constitutive P_*Hpa*I*I*_ promoter. Yet, despite the expression of all tested antibody formats from this promoter, substantial differences in the extracellular levels of these POIs were observed. While the D1.3 scFv was expressed and secreted by all strains tested, the αCRP scFv was expressed and secreted exclusively by the genome-reduced strain *B. subtilis* IIG-Bs27-47-24 but with a higher yield compared to the D1.3 scFv. In contrast, the αCov1 scFv was produced in none of the strains tested, while the αCov2 scFv was produced and secreted by *B. subtilis* IIG-Bs27-47-24 at very low levels, with substantial amounts of this protein remaining associated with the bacterial cell fraction. Of note, the variable nature of the Fv domain implies that the respective amino acid sequences are different in different scFvs. In turn, this seems to have the consequence that different scFvs are secreted with different efficiencies, despite their overall similarity. Potentially, such a bottleneck can be overcome by applying different SPs, as was shown in our present study for secretion of the D1.3 scFab. Such SP swaps presumably lead to a better match of the SP–POI combination with the Sec secretion pathway of *B. subtilis*, for instance through enhanced targeting to the membrane, enhanced membrane translocation, and/or enhanced SP processing.^62−64^ However, other factors such as the mRNA structure and stability, codon usage, and translation rates can also have a profound impact on the production of the different antibody formats, and these factors may explain at least in part the differences in the cellular accumulation and secretion of the analyzed scFvs.^36^

For secretion of the D1.3 scFab, eight different SPs were tested. This resulted in different production and secretion levels. However, the overall yields of the D1.3 scFab remained low compared to the yields of the D1.3 scFv. A noteworthy fundamental difference between scFabs and scFvs is that the scFabs are composed of an N-terminal V_L_–C_L_ domain and a C-terminal V_H_–C_H_1, while scFvs are usually composed of an N-terminal V_H_ fragment and a C-terminal V_L_ fragment. Therefore, despite the fact that both the investigated scFv and scFab are derivates of the D1.3 IgG, the sequence following the SP is different. This may be one explanation why the SP_LipA_ directs effective secretion of the D1.3 scFv, but not of the D1.3 scFab, because it is known that the first residues C-terminally of the signal peptidase cleavage site can have a profound influence on the secretion efficiency of the mature protein.^65^ Another possible explanation for the different expression levels might be scFab degradation by quality control proteases due to improper folding of the C_H_1 domain. In particular, proper folding of the C_H_1 domain is known to require cis–trans prolyl isomerization, which is a rate-limiting step in protein folding that is catalyzed by PPIs.^66^ In addition, the C_H_1 domain requires stabilization by chaperones prior to its association with the C_L_ domain.^20^ As mentioned above, the main extracytoplasmic PPI and chaperone of *B. subtilis*, the lipoprotein PrsA, was shown to be significantly overexpressed in the deployed strain IIG-Bs27-47-24,^59,60^ whereas omission of the disulfide bond between the C_H_1 and C_L_ domains in the scFab was shown to resolve a bottleneck in the maturation of the D1.3 Fab in a previous study.^21^

To determine whether secretion of the D1.3 scFv could be further enhanced, we explored the possibility of using not only SP_LipA_ but also the SP-Pro_Lip_. As shown previously for secretion of the *Escherichia coli* alkaline phosphatase PhoA throughout various *B. subtilis* strains, the SP-Pro_Lip_ module of the *S. hyicus* lipase can effectively facilitate protein secretion to significantly higher levels than only the SP_Lip_ (or any other SP tested) by itself.^45,67,68^ Furthermore, the Pro_Lip_ has previously been used successfully for the secretion of a broad range of different POIs,^69^ including human proinsulin,^70^ the *E. coli* outer membrane protein A (OmpA),^71^ an antigenic portion of the cholera toxin B (CTBp) for surface display,^72^ human growth hormone,^73^ human calcitonin (hCT),^74^ human β1,4-galactosyltransferase 1 (B4GalT-1),^75^ and antibody fragments.^76,77^ Indeed, the SP-Pro_Lip_-D1.3 scFv fusion resulted in higher-level secretion of this scFv than was achieved with the SP_LipA_. However, the Pro_Lip_ remained associated with the D1.3 scFv upon secretion, possibly due to a lack of exoproteases which would be needed for Pro_Lip_ processing in the strains IIG-Bs27-39 and IIG-Bs27-47-24. This result is reminiscent of the incomplete Pro_Lip_ cleavage that was previously observed when the SP-Pro_Lip_-PhoA fusion was expressed in the IIG-Bs27-47-39 strain, whereas complete cleavage of Pro_Lip_-PhoA was observed in the nongenome-reduced protease-proficient control strain *B. subtilis* TS10.^45^ So far, it remains unknown which of the eight *B. subtilis* exoprotease(s) is/are responsible for Pro_Lip_ processing. Conceivably, Pro_Lip_ processing could be facilitated by coexpression of the required exoprotease(s), but this might also lead to degradation of the associated POI.

Cultivation of *B. subtilis* IIG-Bs27-47-24 at the 1 L bioreactor scale allowed purification of the αCRP scFv from the culture supernatant via Ni-NTA affinity chromatography and size exclusion chromatography for further biophysical analyses. The results showed that the αCRP scFv was correctly folded, displaying efficient binding to its cognate antigen CRP and high thermostability compared to other scFvs of common therapeutically applied antibodies.^78^ Importantly, LC-ESI-MS revealed that ∼99% of the detected protein was correctly disulfide-bonded and that merely ∼2.6% of the total product had undergone proteolytic cleavage during fermentation. A previous study has reported a *K*_D_ of 1 × 10^–8^ for the αCRP scFv expressed in *E. coli*, which was determined via surface plasmon resonance spectroscopy.^79^ Our best-fitting computational model based on the kinetic data obtained via BLI suggests a two-step reaction model for the αCRP scFv and human CRP with a *K*_D_ of 5.88 × 10^–7^ and a low χ^2^ value of <0.01% of *R*_max_. This implies that the αCRP scFv undergoes a conformational change during binding to human CRP. Furthermore, given the fact that the αCRP scFv secreted by *B. subtilis* was correctly folded and disulfide-bonded, it seems most likely that the slightly higher *K*_D_ for the αCRP scFv secreted by *B. subtilis* compared to the *K*_D_ measured for this protein upon production in *E. coli* relates to different experimental conditions and techniques that were applied to measure the *K*_D_, rather than the different expression hosts.

## Conclusions and Outlook

In the present study, we report that the genome-reduced *MidiBacillus* strain IIG-Bs27-47-24 is capable of secreting three different scFvs and one scFab. This shows that this strain can be applied as a chassis to secrete antibody fragments. As exemplified for the αCRP scFv, the secreted molecules were properly folded, disulfide-bonded, thermostable, and functionally binding the human CRP antigen. This is fully in line with the results from our previous study, in which we showed a 3,000-fold enhanced secretion of active GLuc by the IIG-Bs27-47-24 strain compared to the TS10 control strain with the full-size genome.^45^ We therefore conclude that the IIG-Bs27-47-24 strain is a high-potential chassis for the effective secretion of disulfide-bonded proteins. For further improvement of this chassis, it will be important to minimize the residual proteolytic activity and further increase the POI production levels. For the latter purpose, the SP-Pro_Lip_ module may be helpful, but effective Pro_Lip_ processing needs to be ensured. Possibly, a simple in vitro protease cleavage step could suffice for this purpose. This might be achieved by insertion of a TEV protease cleavage site between Pro_Lip_ and the POI and treatment with the TEV protease upon affinity purification of the secreted Pro_Lip_-POI.^80^ Alternatively, another specific protease cleaving at the native cleavage site of the used propeptide could be recombinantly expressed in the IIG-Bs27-47-24 chassis strain, such as the SphII metalloprotease (GenBank ID: WP_039646671.1), which is responsible for postsecretional Pro_Lip_ cleavage in *S. hyicus*.^81^ Lastly, it is likely that the fermentation conditions can be further optimized by utilizing different media that meet the specific requirements of the genome-reduced chassis. In any case, while there is certainly room for further improvements, our present study provides a proof of principle that high-quality antibody fragments can be produced with the help of genome-reduced *B. subtilis* strains. This indicates great potential for the production of these important diagnostic and therapeutic tools in bulk and at low cost. Future studies should investigate the wider range of disulfide-bond-containing antibody formats that can be produced with genome-reduced *B. subtilis* strains and how any remaining bottlenecks in their production or secretion by genome-reduced strains can be removed or circumvented.

## Materials and Methods

### Media and Solutions

All media and solutions were prepared using water processed with a Milli-Q Direct Water Purification System (Merck) and sterilized by autoclaving at 121 °C for 15 min. Heat-sensitive medium additives were filter-sterilized.

#### Lysogeny Broth

Lysogeny broth (LB) medium consisted of 10 g/L tryptone (Oxoid by Thermo Fisher Scientific, USA), 5 g/L yeast extract (Difco by Becton, Dickinson and Company), and 10 g/L NaCl.^30^ LB agar contained 1.5% (w/v) agar–agar.

#### Terrific Broth

TB medium consisted of 12 g/L tryptone (Oxoid by Thermo Fisher Scientific), 12 g/L yeast extract (Difco by Becton, Dickinson and Company), and 5 g/L glycerol, as well as 2.31 g/L KH_2_PO_4_ and 12.54 g/L K_2_HPO_4_ which were autoclaved separately.

#### ABB/ABB^+^ (Amazing Bacillus Broth)

ABB medium consisted of 25 g/L soy peptone (Carl Roth), 25 g/L yeast extract (Oxoid), 5 g/L Potato Dextrose Broth (Carl Roth), and 8.20 g/L KH_2_PO_4_ and 12.15 g/L K_2_HPO_4_. ABB^+^ medium was additionally supplemented with 10 g/L sucrose.

#### TQM Medium

1 L of TQM defined medium was always prepared freshly by mixing base solution A (25 g (NH_4_)_2_SO_4_, 3.52 g KH_2_PO_4_, 5.3 g Na_2_HPO_4_ dissolved in 850 mL water, autoclaved), magnesium solution B (2.1296 g MgSO_4_·7H_2_O dissolved in 10 mL water, autoclaved), glutamate solution C (7.35 g l-glutamic acid dissolved in 100 mL water, adjusted to pH = 7.5 with NaOH, sterile filtered), sugar solution D (8 g d-glucose dissolved in 16 mL water, autoclaved), trace element solution E (0.0184 g MnSO_4_·1H_2_O, 0.0206 g ZnSO_4_·7H_2_O, 0.0081 g CuSO_4_·5H_2_O, 0.003480 g CoCl_2_·6H_2_O, 0.0003 g H_3_BO_3_, and 0.00017 g Na_2_MoO_4_ dissolved in 10 mL water, sterile filtered, and protected from light exposure), iron solution F (0.1006 g FeCl_3_·6H_2_O and 0.143 g citric acid dissolved in 1 mL of water, sterile filtered, and protected from light exposure), and calcium solution G (0.0522 g CaCl_2_·2H_2_O dissolved in 10 mL water) in the exact stated order.

#### 4× PAB (Penassay Broth)

4× PAB was prepared by dissolving 35 g of “Penassay Broth” (alternatively named “Antibiotic Medium 3” or “Antibiotic Broth”) (Difco by Thermo Fisher Scientific) in 500 mL of H_2_O.

#### 2× SMM Medium

2× SMM medium consisted of 1 M sucrose, 0.04 M maleic acid, and 0.04 M MgCl_2_, adjusted to pH = 4.6 with NaOH. It is noteworthy that the solution must not change its color to brown upon autoclaving. To ensure this, the medium was prepared in volumes not larger than 250 mL and autoclaved at 121 °C no longer than 15 min.

#### SMMP Medium

SMMP medium was prepared by mixing 4× PAB (50%), 2× SMM medium (45%), and a sterile-filtered solution of 5% (w/v) Bovine Serum Albumin (BSA) in 2× SMM (5%).

#### DM3 Medium

1 L of DM3 medium was prepared from individually prepared watery solutions: 500 mL of 1 M Na-succinate·6H_2_O pH 7.3, 100 mL of 5% (w/v) casamino acids, 50 mL of 10% (w/v) yeast extract, 100 mL of 3.5% (w/v) K_2_HPO_4_ and 1.5% (w/v) KH_2_PO_4_, 200 mL of 4% (w/v) agar, 25 mL of 20% (w/v) d-glucose, 20 mL of 1 M MgCl_2_, and 5 mL of 5% (w/v) BSA.

#### Antibiotics

Unless stated otherwise, media for strains carrying antibiotic resistance markers were supplemented with 100 mg/L ampicillin for *E. coli*, 150 mg/L erythromycin for *E. coli*, 50 mg/L kanamycin (for *E. coli*) or 25 mg/L kanamycin (for *B. subtilis*), 10 mg/L chloramphenicol for *B. subtilis*, or 10 mg/L tetracycline for *B. subtilis*.

### Strain Maintenance and Engineering

#### Culture Conditions

For protein expression studies, three *B. subtilis* chassis strains were used: TS10, IIG-Bs27-39, and IIG-Bs27-47-24 (Table 1), each carrying either an antibody-encoding plasmid or no plasmid for control (Table 1). Unless stated differently, all *B. subtilis* strains were cultured at 37 °C with vigorous shaking at 250 rpm in 20 mL of medium, using 250 mL baffled glass shake flasks (Carl Roth, Germany). For general strain maintenance, *E. coli* and *B. subtilis* were grown in 10 mL of LB medium in 100 mL nonbaffled shake flasks at 37 °C and shaken at 250 rpm.

For protein production experiments with *B. subtilis* grown in LB medium, the different strains were cultured in 250 mL baffled shake flasks containing 20 mL of LB medium with the relevant antibiotics added. Precultures were inoculated from single colonies on LB agar plates and grown for 16–18 h. Main cultures were grown for 18 h. To adapt the strains from the complex to the defined TQM medium, they were passaged at least twice on fresh TQM medium, grown overnight, and reinoculated at an OD_600_ = 0.1. To conserve the adapted strains, cryo-stocks with 20% [v/v] glycerol were prepared from a well-growing culture in the exponential growth phase (OD_600_ ≈ 2). These cryo-stocks were used directly to inoculate the main cultures.

#### Plasmid Construction

All plasmids created within this study were prepared by assembly cloning of two PCR fragments using Phusion High-Fidelity DNA Polymerase (all from New England Biolabs), the GeneArt Seamless Cloning and Assembly Kit (Thermo Fisher Scientific), and Top10 Competent *E. coli* cells (Thermo Fisher Scientific), following the protocol of the manufacturer. In case two oligonucleotides were hybridized to result in one double-stranded DNA insert fragment, both oligonucleotides were mixed in equal volumes, heated to 95 °C, and then cooled down to room temperature again. For constructing pTS1100, part A was amplified from pBSMul1-SP_Epr+1_-GLuc^29^ using the primers TS428/TS429, and part B from the same template using the primers TS430/431. To create the plasmids pTS1101, pTS1102, pTS1106, pTS1107, and pTS1108, the pTS1100 backbone was amplified using the primers TS475/TS476. For pTS1101, the SP_LipA_-D1.3scFv-encoding sequence was amplified from pRBBm117^16^ using the primers TS477/TS478. For pTS1102, the sequences encoding the SP and Pro from the *S. hyicus* lipase (SP-Pro_Lip_) were amplified from pPSPhoA5^45^ using the primers TS432/TS433. For pTS1103, the backbone was amplified from pTS1101 using primers TS471/TS476, and the sequences encoding SP-Pro_Lip_ were amplified from pPSPhoA5 using primers TS479/TS444. For pTS1106, the SP_LipA_-D1.3scFab-encoding sequence was amplified from pRBBm132^83^ using primers TS477/TS478. For pTS1107, the SP-lipA-D1.3scIgG-encoding sequence was amplified from pRBBm136 (unpublished work) using primers TS500/TS478. For pTS1108, the SP_LipA_-αCRPscFv-encoding sequence was amplified from pRBBm230 containing a gene string encoding codon-optimized SP_LipA_-αCRPscFv,^54,84^ using primers TS499/TS478. To create the plasmids pTS1109 and pTS1110, the backbone was amplified from pTS1108 using the primers TS411/TS512, the inset encoding the αCov1 scFv for pTS1109 was amplified from a synthetic DNA fragment (Genewiz, Germany) using the primers TS513/514, and the inset αCov2 for pTS1110 was amplified using the primers TS515/TS516. For pTS1111, the backbone was amplified from pTS1106 using the primers TS517/TS518, and the SP_WapA_-encoding DNA sequence was created by hybridization of the oligonucleotides TS504/TS505. For pTS1112, the backbone was amplified from pTS1106 using primers TS517/TS519, and the SP_AprE_-encoding DNA sequence was created by hybridization of oligonucleotides TS506/TS507. For pTS1113, the backbone was amplified from pTS1106 using the primers TS517/TS520, and the SP_NprE_-encoding DNA sequence was created by hybridization of the oligonucleotides TS508/TS509. For pTS1114, the backbone was amplified from pTS1106 using the primers TS517/TS518, and the SP_Epr_-encoding DNA sequence was created by hybridization of the oligonucleotides TS524/TS525. For pTS1115, the backbone was amplified from pTS1106 using primers TS517/TS522, and the SP_Bpr_-encoding DNA sequence was created by hybridization of the oligonucleotides TS526/TS527. For pTS1116, the backbone was amplified from pTS1106 using the primers TS517/TS523, and the SP_XynA_-encoding DNA sequence was created by hybridization of the oligonucleotides TS528/TS529. For pTS1117, the backbone was amplified from pTS1106 using primers TS517/TS518, and the SP_LytF_-encoding DNA sequence was created by hybridization of oligonucleotides TS530/TS531.

Plasmids were isolated from overnight cultures of *E. coli* using the innuPREP Plasmid Mini Kit (Analytik Jena, Germany). The sequences of plasmid clones were verified by Sanger sequencing^86^ of the cloning site, using the primers JN110/JN111.

All plasmids are listed in Table 1, and all oligonucleotides used as PCR primers or hybridizations are listed in Table S1.

#### Transformation of *B. subtilis*

The strains TS10, IIG-Bs27-39, and IIG-Bs27-47-24 were transformed making use of the introduced supercompetence cassette as described by Rahmer et al.^87^ or via protoplast transformation.

Protoplasts were prepared by growing precultures of the chassis strains in 20 mL of 2 x PAB in 50 mL baffled shake flasks at 37 °C under vigorous shaking overnight. Main cultures were inoculated from the precultures to an OD_600_ of 0.1 and grown in 50 mL of 2× PAB in 500 mL baffled shake flasks until an OD_600_ of 0.4–0.8. The cultures were centrifuged in 50 mL tubes for 10 min with 4,000 *g* at room temperature, the supernatant was discarded, and the cells were resuspended in 10 mL of SMMP medium supplemented with 250 μL of 5% (w/v) lysozyme solution in water. The cell suspensions were incubated in nonbaffled 100 mL shake flasks at 37 °C for 90 min under shaking at 100 rpm, and a protoplast to bacilli ratio of ≥80% was verified via light microscopy. If necessary, the incubation was prolonged. The protoplast cultures were then centrifuged in 10 mL tubes for 15 min with 4,000*g* at room temperature, the supernatant was discarded, and the cells were resuspended in 5 mL of SMMP.

Protoplast transformation was carried out by mixing 500 μL of protoplast suspension with 1000 ng of plasmid DNA, followed by adding 1.5 mL of 40% (w/v) polyethylene glycol 6000 (PEG 6000) in 1× SMM medium. The liquids in the tube were mixed by gentle repeated inversion, followed by rolling the tubes back and forth on a table surface for 2 min. Subsequently, the tubes were centrifuged for 10 min at 4,000*g* at room temperature, the supernatant was discarded, and the cells were resuspended in 1 mL of SMMP medium. To recover the transformed protoplasts, the suspension was incubated for 90 min at 37 °C under shaking with 80 rpm. Afterward, the cells were plated on DM3 plates without antibiotics, whereby ∼300 μL were each carefully spread over the whole surface of one plate. After 3 days of incubation at 30 °C, the cell mass was scratched off from the surface of a plate using a plastic inoculation loop and spread over the whole surface of an LB agar plate supplemented with 25 mg/L kanamycin. The plates were incubated at 37 °C for 24–48 h and checked for colony formation daily. Colonies were restreaked on LB agar plates supplemented with 25 mg/mL kanamycin.

#### Bioreactor Fermentation

Fermentations were performed using a Sartorius Biostat B Plus bioreactor with a 1 L Univessel Glass Column (Sartorius). The pH = 7 was controlled and regulated using 2 M H_2_SO_4_ and 25% ammonia solution. The dissolved oxygen level was set to a minimum of 30% and regulated by an airflow of 3 L/min and via the stirring speed between 350 and 750 rpm. ABB was used as the growth medium for the first preculture, and ABB^+^ was used for the second preculture and the main culture. Fermentations were performed for 24 h.

### Protein Analyses

#### LDS-PAGE of Culture Samples

For the analysis of cell-associated proteins, 100 μL of each culture was centrifuged at 14,000*g* for 2 min at 4 °C in a 2 mL screw-cap tube. The pelleted cells were disrupted by adding 200 μL of 1× NuPAGE lithium dodecyl sulfate (LDS) sample buffer (incl. NuPAGE sample reducing agent) (Thermo Fisher Scientific) and a spatula tip of glass beads with 0.1 mm diameter (Scientific Industries), followed by 2 min of bead beating in a Precellys 24 tissue homogenizer (Bertin Technologies, France). For the analysis of secreted proteins, 100 μL of culture supernatant obtained by centrifugation of the culture at 14,000*g* for 2 min at 4 °C was mixed with 35 μL of 4× NuPAGE LDS sample buffer and 15 μL of 10× NuPAGE sample reducing agent. All samples were subsequently heated to 95 °C for 10 min. Cell lysates were briefly centrifuged, and the supernatant was carefully transferred to a new tube.

For LDS-PAGE, NuPAGE 10% Bis-Tris midi gels (Thermo Fisher Scientific) were loaded with 10 μL of protein from a cell lysate sample or 15 μL of the growth medium sample. Electrophoresis was performed for 1 h at a constant 160 V and maximally 200 mA. For direct visualization of the separated proteins, gels were stained with InstantBlue Coomassie Protein Stain (Abcam).

#### SDS-PAGE with Purified Protein Samples

For analyzing purified protein samples, sodium dodecyl sulfate (SDS) PAGE analyses were performed with either chemically reduced samples or chemically nonreduced samples that had been treated with NEM. To analyze the proteins in a reduced state, the protein sample was diluted to 0.2 mg/mL in its original buffer containing 1× SDS gel loading buffer and 5% β-mercaptoethanol. To analyze the proteins in a nonreduced state, but treated with NEM, NEM was added to the diluted sample to a final concentration of 25 mM and the sample was incubated for 10 min at room temperature, prior to the addition of 5× SDS gel loading buffer (without β-mercaptoethanol) to a final concentration of 1× and 0.2 mg/mL sample protein. The sample was then incubated for 5 min at 95 °C, and different volumes thereof were loaded onto 4–20% Criterion TGX Precast Midi Protein Gels (Bio-Rad Laboratories), i.e., 2 μg, 1 μg, and 0.5 μg. Electrophoresis was performed for 45 min, with a constant 60 mA. For visualization of the separated proteins, gels were stained with Coomassie protein stain.

#### Western Blotting

For Western blotting, the proteins separated by LDS-PAGE were transferred onto Amersham Protran Western blotting membranes using an Invitrogen Power Blotter System (Thermo Fisher Scientific). Membranes were incubated overnight in 5% skim milk solution in 1× phosphate-buffered saline (PBS) including 0.15% Tween 20 (PBS-T) buffer and subsequently washed thoroughly in 1× PBS-T. For protein detection, the membrane was incubated for 1 h with a specific primary antibody diluted 1:5000 in 1× PBS-T and subsequently washed thoroughly in 1× PBS-T. The membrane was then incubated for 1 h with a fluorescently labeled secondary antibody diluted 1:5000 in 1× PBS-T and subsequently washed thoroughly in 1× PBS. All antibodies used are listed in Table 1. Detection of fluorescent signals was performed using an Amersham Typhoon biomolecular imager (Danaher, USA).

#### Protein Purification

ScFv fragments were purified from the culture supernatant obtained upon centrifugation of harvested cultures for 30 min at 4000*g* and 4 °C, followed by filtration with a 4.5 μm pore-sized syringe filter.

The His_6_-tagged scFv fragments were first purified via nickel affinity chromatography using an KTA FPLC system with a HisTrap FF 5 mL column (Cytiva, USA) according to the manufacturer’s manual. Thereby, 450 mL of culture supernatant was mixed with 50 mL of 10× sample buffer (200 mM sodium phosphate, 5 M NaCl, pH = 7.4) and loaded onto the column, which was subsequently washed with 1× washing buffer (20 mM sodium phosphate, 500 mM NaCl, 20–40 mM imidazole, pH 7.4). The POI was then eluted with 1× elution buffer (20 mM sodium phosphate, 500 mM NaCl, 500 mM imidazole, pH 7.4, 250 mM imidazole). The protein content of all flow-through, wash, and elution fractions was examined via LDS-PAGE, and the fractions containing bands of the POI were pooled and concentrated to 1 mL using a Vivaspin 15R 10 kDa MWCO Hydrosart Spin Column (Sartorius, Germany).

The nickel-affinity-purified protein was further purified via size exclusion chromatography using an AΚ̈TA FPLC system with a HiLoad 16/600 Superdex 200 column (Cytiva). The protein content of all flow-through fractions was examined via LDS-PAGE. The fractions containing bands of the POI were pooled and concentrated to 2 mL using a Vivaspin 15R 3000 MWCO Hydrosart Spin Column (Sartorius).

The final concentration of the purified POI was photospectrometrically verified using a NanoDrop (Thermo Fisher Scientific). The purified protein was divided into 50 μL aliquots and snap-frozen in liquid nitrogen.

#### Liquid Chromatography Electrospray Ionization Mass Spectrometry

LC-ESI-MS was employed to measure the molecular weights of the purified proteins. Samples were separated on a BioResolve TMRP mAB polyphenyl column (450 Å, 2.7 μm, 2.1 × 50 mm) (Waters, USA) in 0.1–1% formic acid with an increasing acetonitrile gradient and analyzed with a Q Exactive Plus Orbitrap Mass Spectrometer (Thermo Fisher Scientific). Protein samples (0.5 mg/mL) were analyzed in either an untreated state, a denatured state, or a NEM-trapped denatured state. For the analysis in the untreated state, the protein was diluted in the original protein buffer (20 mM sodium phosphate buffer and 0.5 M NaCl, pH = 7.4), and trifluoroacetic acid (TFA) was added to a final concentration of 0.1%. For denaturation, the protein in the original buffer was mixed with an equal volume of 6 M guanidine-HCl dissolved in 50 mM sodium phosphate buffer, pH = 7.3. After 10 min of incubation at room temperature, TFA was added to a final concentration of 0.5%. For NEM trapping, the samples were treated in the same way as the denatured samples, but additionally, NEM was added to a final concentration of 10 mM prior to incubation. The theoretical molecular weight of the POIs (*M*_theor_) was calculated based on their amino acid sequence plus the C-terminal His_6_ tag using ExPasy ProtParam.^88^ The experimental molecular weight (*M*_exp_) was obtained from mass spectrometry analysis.

#### Biolayer Interferometry

In vitro interactions of αCRP scFv and CRP were analyzed via BLI using an Octet RED384 instrument (ForteBio by Sartorius). Different molar concentrations of CRP (250 nM, 125 nM, 62.5 nM, 15.6 nM, and 7.8 nM) were tested in parallel for binding to the αCRP scFv immobilized on Octet Ni-NTA (NTA) Biosensors (Sartorius) using a dilution of 2 μg/mL. All measurements were performed in HBS Kinetics Buffer (1% heat-inactivated BSA +0.2% Tween 20, 10 mM HEPES, pH = 7.4, 150 mM NaCl) at 30 °C in 96-well microplates. Data were processed using the software Octet Analysis Studio (Sartorius), and a Two State Reaction model was determined as best fitting to the data using the software BIA evaluation (GE Healthcare, USA).

#### Nano-Differential Scanning Fluorimetry

The thermal stability of proteins was analyzed by nanoDSF with a Prometheus NT.48 instrument (NanoTemper Technologies, Germany). Capillaries were filled with 10 μL of scFv protein solution at a concentration of 0.5 mg/mL. The temperature was increased from 20 to 90 °C at a ramp rate of 1 °C/min. The excitation wavelength was 280 nm, and the ratio of emission intensities (Em_350_/Em_330_) was recorded. The fluorescence intensity ratio and its first derivative were calculated with PR.ThermControl (NanoTemper Technologies). The data was further analyzed with MoltenProt (https://spc.embl-hamburg.de/app/moltenprot).

#### Circular Dichroism Spectroscopy

CD spectroscopy was performed using a Chirascan CD spectrometer (Applied Photophysics, UK). CD data were acquired between 280 and 190 nm at 20 °C using a 0.1 cm path length quartz cuvette, every 1 nm with an integration time of 0.5 s. Measurements were repeated three times with a baseline correction. The collected data was processed using the software tools Chirascan Pro-Data Viewer (Applied Photophysics, UK) and BeStSel (https://bestsel.elte.hu/index.php). The direct CD measurements (θ; mdeg) were converted into mean residue molar ellipticity ([θ]_MR_) using the Pro-Data Viewer.

Thermal denaturation of the protein samples was monitored by measuring the CD spectra in the same setup within a temperature range from 20 to 90 °C at a rate of 1 °C/min, using a Peltier Temperature Controller TC125 (Quantum Northwest, USA). The CD data were recorded at every second °C. The *T*_m_ was calculated with the Global3 software (Applied Photophysics) using a one-transition model.

## Data Availability

Plasmid maps and sequences have been deposited on Zenodo with the DOI 10.5281/zenodo.10664122 and are available at https://zenodo.org/records/10664122.

## Supporting Information Available

Tables of all oligonucleotides used in this study and αCRP masses determined by LC-ESI-MS and figure of secretion of D1.3 scFv by *B. subtilis* strains cultured in TQM medium (PDF)
