# Supplementary material for: Toward Antibody Production in Genome-Minimized Bacillus subtilis Strains
Source: ACS Synth Biol. 2025 Feb 27;14(3):740–55. doi: 10.1021/acssynbio.4c00688 (PMC11934139; doi:10.1021/acssynbio.4c00688)
Supplement: Supplementary file 1 — sb4c00688_si_001.pdf [file sb4c00688_si_001.pdf]

Supplemental Material

Towards Antibody Production in Genome-Minimized *Bacillus subtilis* Strains

Tobias Schilling[1], Rebekka Biedendieck[2], Rafael Moran-Torres[3], Mirva J. Saaranen[4], Lloyd W. Ruddock[4], Rolf Daniel[5], and Jan Maarten van Dijl[1]

[1]University of Groningen, University Medical Center Groningen, Department of Medical Microbiology, Hanzeplein 1, P.O. Box 30001, 9700RB Groningen, The Netherlands

[2]Technische Universität Braunschweig, Braunschweig Centre of Systems Biology (BRICS) and Institute of Microbiology, Rebenring 56, 38106 Braunschweig, Germany

[3]Theoretical Biophysics, Humboldt-Universität zu Berlin, Berlin, 10115, Germany

[4]University of Oulu, Faculty of Biochemistry and Molecular Medicine, Protein and Structural Biology Research Unit, Aapistie 7B, 90220 Oulu, Finland

[5]Georg-August-Universität Göttingen, Institute of Microbiology and Genetics, Department of Genomic and Applied Microbiology, Grisebachstr. 8, 37077 Göttingen, Germany

*Correspondence: Jan Maarten van Dijl, University Medical Center Groningen, Department of Medical Microbiology, Hanzeplein 1, 9700RB Groningen, the Netherlands, tel. +31-50-3615187, e-mail: j.m.van.dijl01@umcg.nl

Supplemental Table S1: All oligonucleotides used in this study.

Oligonucleotide Name	5′ → 3′ Sequence
TS428	CTTAAATCGGGCCTACTACCTGTCCCTTGCTG
TS429	CTTTCGTCAGCAAATTCGACCCATCG
TS430	CGAATTTGCTGACGAAAGGGCATCGCGC
TS431	CAGGTAGTAGGCCCGATTTAAGCACACCC
TS432	GGAGGAGATATCATGAAAGAAACAAAACATCAAC
TS433	CGACCTCTAGATTATTTCAGCCCCAGAGC
TS444	CAGTTTAACTTCGTATGGTTTTTTGTCGCTC
TS471	GAAGTTAAACTGCAGGAGTCAGGAC
TS475	TAATCTAGAGGTCGAAATTCAC
TS476	CATGATATCTCCTCCTTATTATG
TS477	GGAGGAGATATCATGAAAAAAGTATTAATGGCTTTC
TS478	TCGACCTCTAGATTAGTGATGGTGATGGTG
TS479	GGAGGAGATATCATGAAAGAAACAAAACATCAAC
TS499	GGAGGAGATATCATGAAAAAAGTATTAATGGCATTT
TS500	GGAGGAGATATCATGAAAAAAGTACTTATGGCATTC
TS504	GGAGATATCATGAAAAAAAGAAAGAGGCGAAACTTTAAAAGGTTCATTGC AGCATTTTTAGTGTTGGCTTTAATGATTTCATTAGTGCCAGCCGATGTACTA GCAGACATCGAGCTCACC
TS505	GGTGAGCTCGATGTCTGCTAGTACATCGGCTGGCACTAATGAAATCATTAA AGCCAACACTAAAAATGCTGCAATGAACCTTTTAAAGTTTCGCCTCTTTCTT TTTTTCATGATATCTCC
TS506	GGAGGAGATATCATGAGAAGCAAAAAATTGTGGATCAGCTTGTTGTTTGC GTTAACGTTAATCTTTACGATGGCGTTCAGCAACATGTCTGCGCAGGCTGC CGACATCGAGCTCACC
TS507	GGTGAGCTCGATGTCGGCAGCCTGCGCAGACATGTTGCTGAACGCCATCGT AAAGATTAACGTTAACGCAAACAACAAGCTGATCCACAATTTTTTGCTTCTC ATGATATCTCCTCC
TS508	GGAGGAGATATCATGGGTTTAGGTAAGAAATTGTCTGTTGCTGTCGCTGCT TCGTTTATGAGTTTATCAATCAGCCTGCCAGGTGTTCAGGCTGCTGACATC GAGCTCACC
TS509	GGTGAGCTCGATGTCAGCAGCCTGAACACCTGGCAGGCTGATTGATAAACT CATAAACGAAGCAGCGACAGCAACAGACAATTTCTTACCTAAACCCATGAT ATCTCCTCC
TS511	TAATCTAGAGGTCGAAATTCACCTC
TS512	TGCAGATGCACCAGATGG

TS513	TCTGGTGCATCTGCACAAGTGCAGCTCGTTGAA
TS514	TCGACCTCTAGATTAATGATGGTGGTGATGATGAGC
TS515	TCTGGTGCATCTGCACAAGTTCAGCTTGTAGAATC
TS516	TCGACCTCTAGATTAATGATGGTGGTGATGATG
TS517	GACATCGAGCTCACCCAGT
TS518	TTTCATGATATCTCCTCCTTATTATGTAAATC
TS524	TAAGGAGGAGATATCATGAAAAACATGTCTTGCAAACTTGTTGTATCAGTCACTCTGTTTTTCAGTTTTCTCACCATAGGCCCTCTCGCTCATGCGCAAGACATCGAGCTCACC
TS525	ggtgagctcgatgtcTTGCGCATGAGCGAGAGGGCCTATGGTGAGAAAACTGAAAAACAGAGTGACTGATACAACAAGTTTGCAAGACATGTTTTTCATGATATCTCCTCCTTA
TS526	TAAGGAGGAGATATCATGAGGAAAAAAACGAAAAACAGACTCATCAGCTCTGTTTTAAGTACAGTTGTCATCAGTTCACTGCTGTTTCCGGGAGCAGCCGGGGCAgacatcgagctcacc
TS527	ggtgagctcgatgtcTGCCCCGGCTGCTCCCGGAAACAGCAGTGAACTGATGACAACTGTACTTAAAACAGAGCTGATGAGTCTGTTTTTCGTTTTTTTCCTCATGATATCTCCTCCTTA
TS528	TAAGGAGGAGATATCATGTTTAAGTTTAAAAAGAATTTCTTAGTTGGATTATCGGCAGCTTTAATGAGTATTAGCTTGTTTTCGGCAACCGCCTCTGCAgacatcgagctcacc
TS529	ggtgagctcgatgtcTGCAGAGGCGGTTGCCGAAAACAAGCTAATACTCATTAAAGCTGCCGATAATCCAACTAAGAAATTCTTTTTAAACTTAAACATGATATCTCCTCCTTA
TS530	TAAGGAGGAGATATCATGAAAAAGAAATTAGCAGCAGGGCTGACAGCATCTGCGATTGTCGGCACAACTTTAGTAGTGACACCAGCTGAAGCAGCAgacatcgagctcacc
TS531	ggtgagctcgatgtcTGCTGCTTCAGCTGGTGTCACTACTAAAGTTGTGCCGACAATCGCAGATGCTGTCAGCCCTGCTGCTAATTTCTTTTTCATGATATCTCCTCCTTA
JN510	GTGGTTGCCGGAAAGAGCGAAAATGCCTC
JN511	GGAGCCTTTAATTGTATCGGTTTATCAGCTTGC

Supplemental Table S2: αCRPscFv masses determined by LC-ESI-MS.

Mass [Da]	Δ_{Mass} [Da]	Fractional Abundance [%]	Putative Molecule
**Untreated Sample**			
29797.20	-0.77	22.18	αCRPscFv DSB
29779.64	-18.33	22.05	αCRPscFv DSB - 1 H_2O
29780.03	-17.94	21.6	αCRPscFv DSB -1 H_2O
29817.12	19.15	6.29	αCRPscFv DSB + 1 H_2O/ NH_4^+
Other minor species		20.93	αCRPscFv DSB + common ion adducts
16329.67	-2.22	1.74	αCRPscFv DSB C-terminal truncated fragment
16332.68	0.79	1.1	αCRPscFv DSB C-terminal truncated fragment
**Denatured Sample**			
29779.57	-18.40	42.05	αCRPscFv DSB -1 H_2O
29797.23	-0.74	23.06	αCRPscFv DSB
29817.13	19.16	6.16	αCRPscFv DSB + 1 H_2O/ NH_4^+
Other minor species		23.44	αCRPscFv DSB + common ion adducts
16329.78	-2.11	1.92	αCRPscFv DSB C-terminal truncated fragment
16332.59	0.70	1.08	αCRPscFv DSB C-terminal truncated fragment
**Denatured + NEM Treated Sample**			
29779.69	-18.28	41.1	αCRPscFv DSB - 1 H_2O
29797.04	-0.93	23.14	αCRPscFv DSB
29816.88	18.91	5.74	αCRPscFv DSB + 1 H_2O/ NH_4^+
Other minor species		22.57	αCRPscFv DSB + common ion adducts
16329.83	-2.06	1.5	αCRPscFv DSB C-terminal truncated fragment
29922.12	124.15	0.94	αCRPscFv + 1 NEM (unspecific side reaction)

DSB, disulfide-bonded

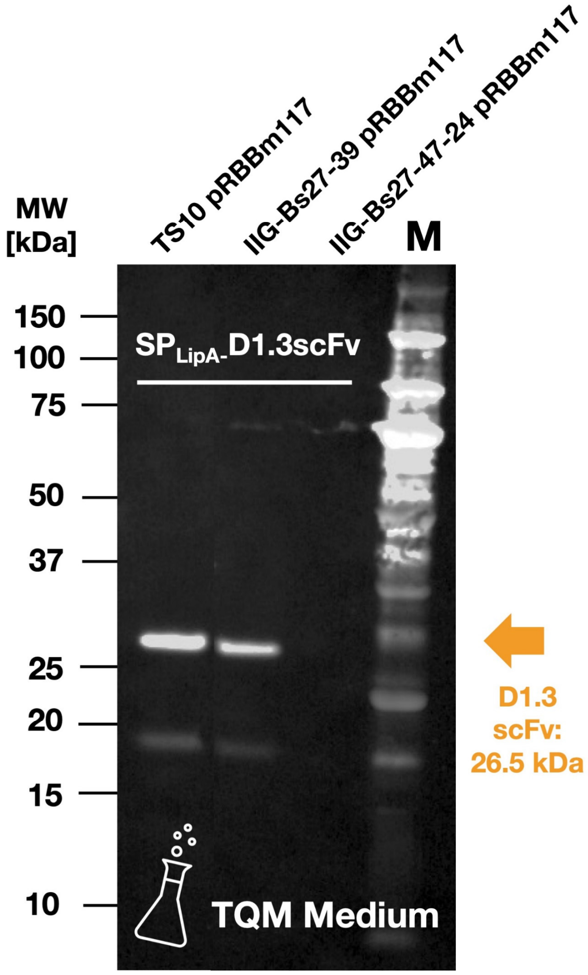

Supplemental Figure S1: Secretion of D1.3 scFv by *B. subtilis* strains cultured in TQM Medium. Western blots of the culture supernatant fractions from different *B. subtilis* strains secreting D1.3 scFv upon growth in TQM medium. Secretion of the D1.3 scFv was visualized with a primary antibody against the C-terminally attached His_6-tag. The colored arrows indicate the expected molecular size in kDa of the respective proteins of interest. The image represents a higher exposed version of a part of Figure 3B to visualize the bands of ±18 kDa with higher mobility in LDS-PAGE compared to the full-size secreting D1.3 scFv.
